# Role of Anthocyanins in the Interaction between Salivary Mucins and Wine Astringent Compounds

**DOI:** 10.3390/foods12193623

**Published:** 2023-09-29

**Authors:** Bárbara Torres-Rochera, Elvira Manjón, María Teresa Escribano-Bailón, Ignacio García-Estévez

**Affiliations:** Department of Analytical Chemistry, Nutrition and Food Science, Universidad de Salamanca, E37007 Salamanca, Spain; barbara.torres@usal.es (B.T.-R.); elvira87@usal.es (E.M.); igarest@usal.es (I.G.-E.)

**Keywords:** high-molecular-weight salivary proteins, phenolic compounds, ITC, astringency, copigmentation

## Abstract

Wine astringency is a very complex sensation whose complete mechanism has not been entirely described. Not only salivary proline-rich proteins (PRPs) are involved in its development; salivary mucins can also play an important role. On the other hand, it has been described that anthocyanins can interact with PRPs, but there is no information about their potential role on the interactions with mucins. In this work, the molecular interactions between salivary mucins (M) and different wine phenolic compounds, such as catechin (C), epicatechin (E) and quercetin 3-*β*-glucopyranoside (QG), as well as the effect of the anthocyanin malvidin 3-*O*-glucoside (Mv) on the interactions with mucins, were assessed by isothermal titration calorimetry (ITC). Results showed that the interaction between anthocyanin and mucins is stronger than that of both flavanols analyzed, since the affinity constant values were 10 times higher for anthocyanin than for catechin, the only flavanol showing interaction in binary assay. Moreover, at the concentration at which polyphenols are usually found in wine, flavonols seem not to be involved in the interactions with mucins. These results showed, for the first time, the importance of wine anthocyanins in the mechanisms of astringency involving high-molecular-weight salivary proteins like mucins.

## 1. Introduction

As a consequence of climate change, grape harvesting is performed when grapes have not yet reached phenolic maturity [1]. Therefore, the sensory properties of wine are affected, such as color and astringency, which are two of the most important organoleptic attributes for red wine quality since phenolic compounds play an important role in these two organoleptic features.

The red color of anthocyanins is due to the flavylium cation, only stable at acidic pH (pH ≤ 2). When the pH increases, the flavylium cation is involved in different equilibrium reactions producing mainly non-colored forms of anthocyanin. The association of anthocyanins with other compounds, in a phenomenon called copigmentation, shifts this equilibrium towards the flavylium cation, thus leading to an improvement in the chemical and colorimetric stability of the anthocyanin. Hence, the intense red color of young red wines (pH *ca.* 3.6) is mostly due to the interactions between the flavylium cation and other wine compounds such as flavanols, flavonols and phenolic acids [2,3]. When physical-chemical studies of the interaction flavonol:anthocyanin are performed, it is observed that association constants are higher than in the case of flavanols. However, their quantity in wine is much lower than the flavanol content, so major interactions between flavanols and anthocyanins are envisaged [4]. Among flavanols, it is well known that (−)-epicatechin is a better copigment than (+)-catechin due to its B ring conformation [5].

Astringency is a sensory experience perceived when consuming certain foods and drinks such as wine, resulting in a sensation of dryness, rugosity or roughness that is generated in the mouth. The interaction of wine phenolic compounds, such as flavanols and flavonols, with salivary proline-rich proteins (PRPs) is the main mechanism related to astringency sensation [6,7]. This leads to protein:polyphenol complexes that can precipitate, thus resulting in a loss of lubrication in the oral cavity [8]. However, it is a very complex sensation whose complete mechanism has not been totally described. It has also been suggested that anthocyanins can also be considered to understand astringency perception, since it has been proved that they can form complexes with the PRPs [9]. Furthermore, a synergic effect of flavanol:anthocyanin mixtures towards the interaction with PRPs when compared to the individual polyphenol has also been observed [10].

PRPs are the main proteins studied to explain astringency. However, there are other types of proteins in saliva such as mucins, which are high-molecular-weight glycoproteins (500 to 20,000 kDa), that are present at the highest concentration in unstimulated whole human saliva (20–30% of the total protein) [11,12,13] and whose relationship with the development of astringency has been barely studied. Mucins are highly glycosylated, consisting of 80% carbohydrates, primarily *N*-acetylgalactosamine, *N*-acetylglucosamine, fucose, galactose, and sialic acid (*N*-acetylneuraminic acid) and traces of mannose and sulfate [14]. Moreover, mucins are attracting more interest for their biological properties and for their involvement in lubrication, hydration and protection of the oral cavity [15,16]. It has been already described that mucins can also interact with food polyphenols, which may affect their lubricating function, so these proteins could also play an important role in astringency [17,18,19] perception [16,17,18,19]. Even though the importance of mucins in oral lubrication is well documented, the study of the role of salivary mucins in the development of astringency is still lacking. Therefore, a more profound study of the interactions between these proteins and wine phenolic compounds is important to learn more details about the factors that could affect the mechanisms of astringency sensation, which, in turn, is very relevant for the acceptable wine quality.

Isothermal titration calorimetry (ITC) is an excellent technique to characterize protein binding to flavanols [10,20,21,22], even when the interaction is weak [10,20,21,22]. The calorimeter detects whether the heat given off by the interaction is exothermic or endothermic and, due to the enthalpy change that these interactions imply, the thermodynamics of protein:ligand interactions and the types of forces involved can be studied [23]. Moreover, this technique is particularly attractive because it does not involve any chemical modification or immobilization of either interacting species, which allows approaching wine tasting at a molecular level. However, the interpretation of the results from this technique can be difficult because polyphenol:protein interaction does not follow the classical “lock and key” model [24,25], and it is necessary to fit the data following one model that contemplates multiple independent binding sites [26].

To obtain new insights about the role of mucins in astringency sensation, the main objective of this work was to study and to characterize, by ITC and at an acidic pH of 3.6, in which wine is found, the interaction of salivary mucins (M) with different wine phenolic compounds (catechin (C), epicatechin (E) and quercetin 3-*β*-glucopyranoside (QG)). Recently, it has been reported that cyaniding 3-*O*-glucoside is able to interact with mucins mainly when oxidized to its quinone form and that that interaction is driven by hydrogen bond and van der Waals forces, according to ITC studies [27]. This points out a potential role of anthocyanins in astringency development. However, the main anthocyanin in most wines is malvidin 3-*O*-glucoside, which, in turn, is involved in the interaction with other phenolic compounds present in wines due to copigmentation. This way, this research further explored the knowledge about the mechanisms of wine astringency by including different phenolic compounds and mainly by studying the role of the main anthocyanin in wines (malvidin 3-*O*-glucoside) on the interactions with salivary mucins. Furthermore, and due to the significance of the copigment interactions in wine, it was assessed for the first time whether the interactions between mucins and flavanols and mucins and flavonols are affected, in turn, by the copigmentation interaction. To achieved this, the ternary interactions involving malvidin 3-*O*-glucoside (Mv), the phenolic compound (flavanols (C and E) or flavonol QG) and salivary mucin were also deeply characterized by ITC.

## 2. Materials and Methods

**Chemicals.** (+)-catechin hydrate (≥98%) (C), (−)-epicatechin (≥90%) (E) and mucin (M) from bovine submaxillary glands were acquired from Sigma-Aldrich (St. Louis, MO, USA). Quercetin 3-*β*-glucopyranoside (≥99%) (QG) was purchased from Cymit Quimica (Barcelona, Spain). Ultrapure water came from a water purification system of MiliQ Gradient (Millipore, Billerica, MA, USA). The malvidin 3-*O*-glucoside pigment was obtained by isolation in the laboratory as it is explained below.

**Isolation of Malvidin 3-*O*-Glucoside (Mv).** Skins of *Vitis vinifera* cv Tempranillo grapes were used as source of Mv for isolation. The extraction of the grape skins was performed by using acidic methanol (methanol/HCl 0.5 N; 95:5 *v/v*) as described in García-Estévez et al. [28]. To purify the Mv, a Sephadex LH-20 (Sigma-Aldrich, St. Louis, MO, USA) column was employed. This column was 40 cm high and had a diameter of 3.5 cm, which was previously conditioned using acidic water as eluent (HCl 0.1 M, pH 1.0) before the extract was loaded, as described in García-Estévez et al., with some minor modifications [29]. Skin extract was loaded into the column and then the elution was performed using the aqueous HCl solution. Mv eluted in the first fraction collected (*ca*. 20 mL). The process was repeated to obtain several fractions, and their purity was checked by HPLC-DAD-MS, showing values of purity >95%, and then collected and freeze-dried to provide a dark reddish purple powder.

**Isothermal Titration Calorimetry Assays.** The thermodynamic parameters associated with binary and ternary mucin:polyphenol interactions were obtained from the ITC experiments using a MicroCal PEAQ-ITC system (Malvern, Worcestershire, UK). Studies were performed at 298 K (25 °C), which is the temperature of the mouth when wine is tasted. In all cases, the phenolic compound (PC) solution was charged into the injection syringe while the mucin was put into the 0.2 mL sample cell of the calorimeter, and the content of the sample cell was constantly stirred at 1200 rpm. All the assays consisted of a sequence of 19 injections of 2 µL each, with the time of the injection duration and the time between successive injections set as 2 s and 250 s, respectively. All solutions were prepared at wine pH (pH 3.6). The mucin solution was prepared at 500 nM and PC solutions were prepared at the following concentrations: 250 µM QG, 500 µM E and 2000 µM C. Mv solutions were prepared at the same concentration of each PC and 1:1 PC:Mv mixtures were also prepared at those concentrations (250 µM QG:250 µM Mv; 500 µM E:500 µM Mv and 2000 µM C:2000 µM Mv). These concentrations were selected within the usual concentrations in wine to achieve the saturation of the process and to record sufficient energy signal. Blank experiments, where acidic water (pH 3.6) filled the sample cell, were also performed at all concentrations for each compound previously mentioned. All experiments were performed in triplicate. Data treatment was carried out using software AFFINimeter (Version 1.2.3) (Software for Science Developments, Santiago de Compostela, Spain), which allowed the use of an independent sites model with two different types of sites for the interaction (two sets of sites) to obtain the fitting curve (enthalpy change vs. molar ratio). From that fitting, the binding apparent constant (*K*), the Gibbs free energy (Δ*G*), the enthalpy change (Δ*H*) and the entropy component (−T·Δ*S*) were calculated.

**Statistical Analysis.** The statistical significance of the differences between the results were assessed by using one-way analysis of variance (ANOVA) and post hoc Tukey test employing software GraphPad Prism (Version 5.02). Differences were considered statistically significant at *p* < 0.05.

## 3. Results and Discussion

ITC is an interesting technique used to obtain the thermodynamic parameters that govern protein:ligand interactions [24,25,30]. Binary interactions between different PC, i.e., flavanols (C, E) or a flavonol (QG) with mucin were studied by this technique to determine whether these phenolic compounds could interact with high-molecular-weight salivary glycoproteins and whether that way these proteins could participate in the astringency perception. Furthermore, ternary interactions, flavanol–anthocyanin or flavonol-anthocyanin with mucin, were also studied to figure out whether a previous interaction established between the phenolic compounds, the so-called copigmentation interactions that occur in the wine matrix, could affect the interactions with the protein.

In this study, C and its isomer E were chosen because flavanols are frequently the most abundant phenolic compounds found in red wines, and QG was used because it is well known that flavonols easily interact with anthocyanins establishing copigmentation adducts due to their quasi-planarity. As has been mentioned in Section 2, the concentrations assayed for the ITC experiments were selected within the usual concentrations found in wine to record sufficient energy signal until process saturation was reached.

The model that best fit the isothermal data considered two sets (Set 1 and Set 2) and independent sites. The mechanisms of binding were evaluated by comparing the change in enthalpy (Δ*H*) and in the entropic component (−T·Δ*S*) of the systems. When the process is driven by enthalpy, which means negative values of Δ*H*, interactions are associated with hydrogen bonding [21], which may occur in a first step when polyphenol and protein interact to form the complexes and/or between those complexes in a second step to form aggregates. However, the process is driven by entropy when negative values of –T·Δ*S* are obtained, and then, the main forces are hydrophobic interactions [21] that lead to the displacement of water molecules. In all cases, the negative values of the change in Gibbs free energy (Δ*G*) indicate that the resulting interactions are spontaneous. Furthermore, the binding constants (*K*) were also obtained; the higher the *K* value*,* the stronger the protein:polyphenol interaction [31].

### 3.1. Interaction Assays Corresponding to Catechin System

**Figure 1:**
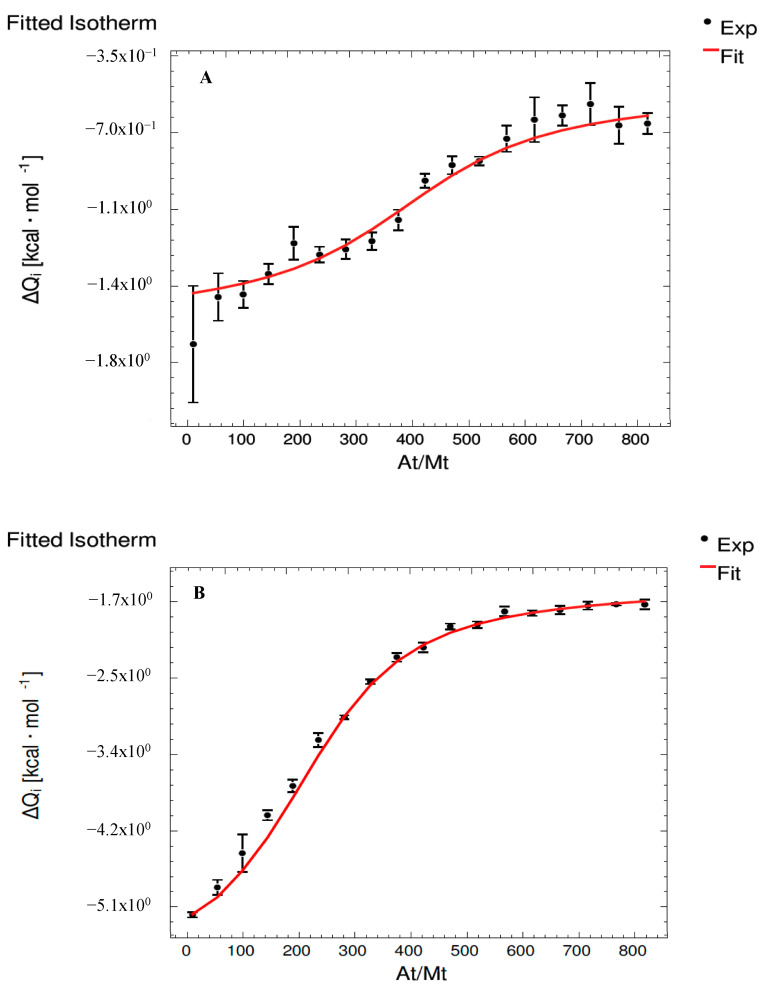

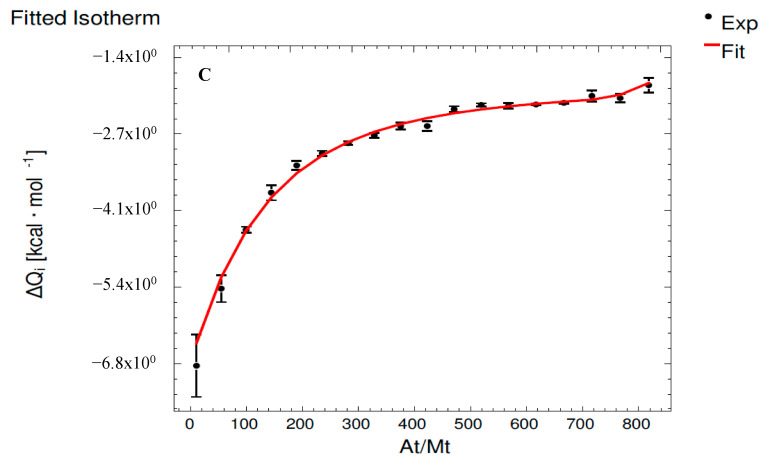
Isotherm fitting for (**A**) MC, (**B**) MMv_2000_ and (**C**) MMvC systems. At/Mt: ratio M/C, ratio M:Mv_2000_ and ratio M/Mv:C, respectively.

Figure 1 shows the isotherm fitting for the binary interactions (MC and MMv_2000_, Figure 1A,B, respectively) and the corresponding ternary interaction (MMvC, Figure 1C) systems. The thermodynamic parameters obtained (*K*, Δ*G*, Δ*H* and −T·Δ*S*) for each system are shown in Table 1. In all cases, it was observed that the released energy indicated spontaneous processes (Δ*G* values were all negative), so both C and Mv are able to interact with M both separately or when they are mixed. However, exothermic and endothermic processes occur depending on the interaction, as is explained below. In the literature, similar results have been reported for the interaction of individual flavanols and other proteins such as salivary proteins [25], PRPs [10] and other model proteins such as poly-(L-proline) [22] and bovine serum albumin [32]. However, as can be seen in Table 1, the most negative Δ*G* values were found for the MMv_2000_ interaction for Set 1 and Set 2 (−1.43 × 10^4^ and −1.22 × 10^4^, respectively), which indicates a higher affinity of Mv to M when compared to C, as the highest binding constant (3.22 × 10^10^ and 8.92 × 10^8^ M^−1^) for the MMv_2000_ interaction also pointed out. Thus, it seems that the anthocyanin has a higher affinity to mucins than catechin, which might indicate that pigments could play an important role in the astringency development involving high-molecular-weight salivary proteins. MMvC ternary interaction showed values indicating the weakest interaction for both compounds, C and Mv, and in both sets (see *K* values of MMvC (C) and MMvC (Mv) in Table 1). Moreover, when Mv and C were together in the solution, both compounds showed very similar affinity towards M, whereas, as explained before, when these compounds were assayed isolated, Mv showed a much stronger interaction than C.

Regarding the driving forces involved in these processes, it can be observed that in both binary interactions (MC and MMv), both types of forces, hydrophobic interactions and hydrogen bonds (H-bonds), take place, since both Δ*H* and −T·Δ*S* show negative values [33] (Table 1). From previous studies performed involving salivary proteins and flavanols, it is widely known that this interaction usually involves both types of bonds, i.e., hydrophobic interactions and hydrogen bonding [22,25,34,35]. Our results show that anthocyanins interact in a similar way with mucins, which gives rise to new information about the mechanism that takes place between high-molecular-weight proteins and anthocyanins. However, when the Mv:C mixture is assayed towards the interaction with M, the forces involved in the interaction change when compared to the corresponding binary interaction. Also, within each set of sites, the forces involved in the interaction of Mv with M and in that between C and M are different (Table 1). Indeed, in Set 1, the interaction between C and M (see MMvC (C)) is mainly driven by hydrophobic interactions (Δ*H* > 0 and −T·Δ*S* < 0), whereas Mv (see MMvC (Mv)) seems to bind to M mainly thought H-bonds (Δ*H* < 0 and −T·Δ*S* > 0). Likewise, the contrary effect occurs in Set 2, in which C interacts with M by H-bonds, whereas the M:Mv interaction is driven by hydrophobic forces.

Therefore, the studied interactions might occur by hydrophobic bonds between the planar surfaces of the polyphenol and the ring planes of the mucin amino acids, whereas hydrogen bonds may occur by polar interactions among the carbonyl and amino groups of the mucin and the hydroxyl groups on the catechin. This is in agreement with studies reported in the literature about flavanol:salivary protein interaction that show that hydrophobic interactions (π-π bonding) occur between heterocyclic proline rings of PRPs and the planar surfaces of the aromatic rings of flavanols, whereas hydrogen bonds happen between hydroxyl groups of flavanols and the carbonyl and amino groups of the protein residues [21,22].

Thus, these results point out that the interaction between Mv and C that occurs when these compounds are in the same solution hinder the interaction of both compounds with M, so the formation of the copigmentation complexes Mv:C complicates the interaction with the protein. This could be due to both lesser availability of the interaction points of these compounds due to the previous formation of the copigmentation complex and a potential steric hindrance of this complex that does not allow the interaction with mucin. These results indicate that the presence of Mv in the same concentration as that of C reduces the interaction of this flavanol with mucins, which could be related to the changes in the way in which the interaction occurs, as the change in the forces involved indicates. This may suggest an indirect role of the anthocyanin in the interaction between flavanols and high-molecular-weight salivary proteins, highlighting the importance of wine pigments for the mucin-related mechanisms of astringency.

### 3.2. Interaction Assays Corresponding to Epicatechin System

**Figure 2:**
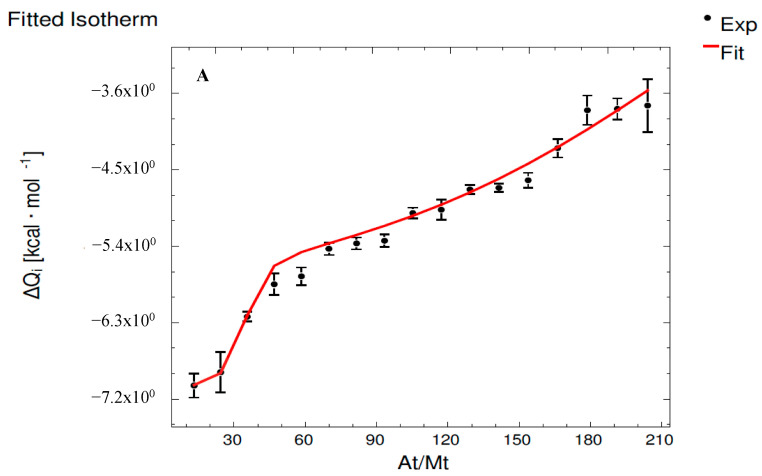

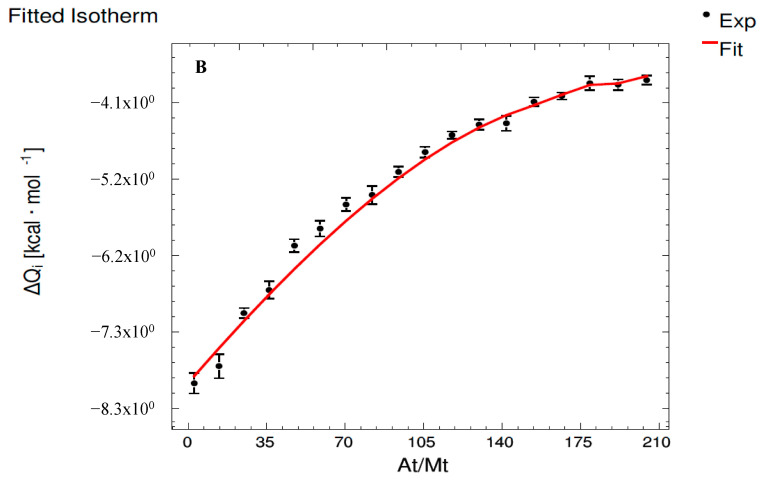
Isotherm fitting for (**A**) MMv_500_ and (**B**) MMvE systems. At/Mt: ratio M:Mv_500_ and ratio M/Mv:E, respectively.

Figure 2 shows the isotherm fittings for MMv_500_ (Figure 2A) and MMvE (Figure 2B) systems. The binary interaction between M and E did not release or consume enough heat to be detected by the microcalorimeter, so no fitting can be performed. However, according to previous ITC studies involving E [10], this flavanol spontaneously interacts with basic PRPs, but no interaction is observed using this technique between E and acidic PRPs, so it seems that the interaction between E and salivary proteins is highly dependent on the structure of the protein involved.

In the case of the interaction between M and Mv and in the ternary interaction, as can be observed in Table 2, the released energy indicates spontaneous processes (Δ*G* < 0). Moreover, as for the C system, Mv presents a higher binding affinity towards M than E, which is also observed in the case of PRPs [10]. This points out, again, the importance that wine pigments may have on astringency development.

However, in this case, the binary interaction between Mv and M is less strong than the ternary interaction, where both compounds, Mv and E, show higher affinity constants in the interaction with M than in that with the isolated Mv. Thus, the presence of both E and Mv in the solution enhances the ability of those compounds to interact with M, which is more noticeable for E since it is not able to interact with M when it is isolated. This is in agreement with previous studies that showed that Mv:E mixtures have the highest affinity towards other salivary proteins, namely PRPs [10], so it seems that, contrarily to that observed for C, the involvement of E and Mv in the copigmentation adducts may favor the interaction with salivary proteins, both low- and high-molecular-weight proteins. That way, anthocyanins can have a strong indirect effect on the role of E on the wine astringency development.

Moreover, the driving forces involved in these systems can be deduced from these ITC results (Table 2). As previously indicated, both forces, hydrophobic and H-bonds, are involved in the binary interaction between M and Mv (Δ*H* < 0 and −T·Δ*S* < 0). Also, as in the case of C, when we observe the ternary interaction MMvE, the forces involved are different from those in the binary interaction MMv. However, in this case, the forces involved in the interaction between M and E and Mv in the ternary process are the same within each set of sites. In Set 1, results show that hydrophobic interactions (Δ*H* > 0 and −T·Δ*S* < 0) are predominant, whereas H-bonds (Δ*H* < 0 and −T·Δ*S* > 0) are the main driving forces for Set 2. These results indicate that the formation of the copigmentation adducts Mv:E modifies the way in which these compounds interact with M, regarding both the strength of the interaction and the type of forces involved. As for that previously reported for other salivary proteins of lower molecular weight than that of mucin, Mv seems to drive the interaction, and its presence seems to favor the interaction of E with M, which could be explained by two reasons. On the one hand, it could be explained since the formation of Mv:E copigmentation complexes allows the molecules the establishment of interactions with mucins through more prone interaction points, which, in turn, explains the modifications in the forces involved in the interaction. Indeed, it has been reported for PRPs that when E is isolated, A and mostly B rings from this flavanol are involved in the interaction, whereas in the presence of Mv:E, both phenolic compounds start to interact with PRPs through their ring B, which involved not only the phenolic parts of the molecules but also the methyl groups of the Mv B ring, as well as with the glucose moiety [10]. On the other hand, previous studies performed to assess the copigmentation interactions between flavanols and Mv by molecular dynamic simulations [36] showed that Mv:E are smaller than Mv:C adducts, since the distance between Mv and E in the adduct is shorter (4.5 Å) than that between Mv and C (4.8 Å). Thus, the Mv:E adducts might have less steric hindrance, which would favor their interaction with mucin.

### 3.3. Interaction Assays Corresponding to QG System

As in the case of E, the interactions in which QG is involved do not release or consume enough heat to be detected by the microcalorimeter, so it can be deduced that, at the concentration at which this compound is usually found in wine, it is not able to interact with mucin. This points out that flavonols might not be involved in the mechanism of astringency related to high-molecular-weight proteins, and the astringency reported for these phenolic compounds [37] may be explained by their ability to interact with PRPs [37] and with oral cells [38]. Also, when Mv is included in the system, no interaction is observed in the ternary assay, which points out that, in this case, the presence of the anthocyanin does not modify or favor the interaction between QG and M. This could be explained since, among the assayed phenolic compounds, the most stable interaction and the highest binding affinity is found, by far, between QG and Mv [36], so it seems that the strong interaction between QG and Mv hinders the interaction of those compounds with M.

## 4. Conclusions

In order to obtain insights about the potential role of mucins in wine astringency sensation, the molecular interactions between salivary mucins (M) and different wine phenolic compounds, such as catechin (C), epicatechin (E) and quercetin 3-*β*-glucopyranoside (QG), as well as the effect of copigmentation involving malvidin 3-*O*-glucoside (Mv) on the interactions with mucin, were assessed by ITC. Results pointed out the importance of anthocyanins in the interactions with high-molecular-weight salivary proteins such as mucins, since Mv is a phenolic compound showing, when isolated, the strongest affinity towards mucin. On the other hand, it seems that flavonols, such as QG, are not involved in the interactions with this type of protein at the concentration that they are usually found in red wines. Copigmentation seems to be also relevant for the interactions between phenolic compounds and mucins, since the presence of Mv in the solutions modifies the intensity and the characteristics (regarding the forces involved) of the interactions between flavanols and mucins, although in a different way depending on the flavanol structure. Thus, anthocyanins could play an important role, both directly and indirectly, in the astringency development involving high-molecular-weight salivary proteins like mucins.

## Figures and Tables

**Table 1:** Thermodynamic parameters for binary (M:C and M:Mv) and ternary (M:Mv:C) interactions.

**Set 1**	**MC**	**MMvC (C)**	**MMvC (Mv)**	**MMv_2000_**
*K*_1_ (M^−1^)	(1.92 ± 0.21) × 10^9^ b	(5.11 ± 0.21) × 10^7^ c	(1.97 ± 0.08) × 10^7^ c	(3.22 ± 0.03) × 10^10^ a
Δ*H*_1_ (cal·mol^−1^)	(−1.02 ± 0.02) × 10^3^ b	(1.00 ± 0.06) × 10^5^ a	(−4.03 ± 0.13) × 10^5^ c	(−6.01 ± 0.01) × 10^3^ b
Δ*G*_1_ (cal·mol^−1^)	(−1.26 ± 0.13) × 10^4^ a,b	(−1.05 ± 0.04) × 10^4^ a	(−9.93 ± 0.40) × 10^3^ a	(−1.43 ± 0.01) × 10^4^ b
−T·Δ*S*_1_ (cal·mol^−1^)	(−1.16 ± 0.14) × 10^4^ b	(−1.10 ± 0.06) × 10^5^ c	(3.93 ± 0.13) × 10^5^ a	(−8.30 ± 0.14) × 10^3^ b
**Set 2**	**MC**	**MMvC (C)**	**MMvC (Mv)**	**MMv_2000_**
*K*_2_ (M^−1^)	(6.09 ± 0.08) × 10^7^ b	(1.00 ± 0.01) × 10^7^ c	(1.02 ± 0.02) × 10^7^ c	(8.92 ± 0.20) × 10^8^ a
Δ*H*_2_ (cal·mol^−1^)	(−1.55 ± 0.10) × 10^2^ b	(−1.13 ± 0.01) × 10^5^ d	(9.10 ± 0.01) × 10^4^ a	(−1.36 ± 0.01) × 10^3^ c
Δ*G*_2_ (cal·mol^−1^)	(−1.06 ± 0.01) × 10^4^ b	(−9.53 ± 0.09) × 10^3^ a	(−9.54 ± 0.19) × 10^3^ a	(−1.22 ± 0.03) × 10^4^ c
−T·Δ*S*_2_ (cal·mol^−1^)	(−1.04 ± 0.01) × 10^4^ b	(1.04 ± 0.01) × 10^5^ a	(−1.01 ± 0.01) × 10^5^ c	(−1.08 ± 0.03) × 10^4^ b

MMvC (C): the interaction parameters of C in the ternary system; MMvC (Mv): the interaction parameters of Mv in the ternary system. Different letters in each row indicate statistical differences (*p* < 0.05) according to post hoc Tukey test.

**Table 2:** Thermodynamic parameters for binary (M:E and M:Mv_500_) and ternary (M: Mv:E) interactions.

**Set 1**	**ME**	**MMvE (E)**	**MMvE (Mv)**	**MMv_500_**
*K*_1_ (M^−1^)	-	(9.29 ± 0.35) × 10^8^ a	(6.97 ± 0.15) × 10^8^ b	(6.20 ± 1.70) × 10^7^ c
Δ*H*_1_ (cal·mol^−1^)	-	(2.03 ± 0.10) × 10^5^ a	(1.37 ± 0.10) × 10^5^ b	(−6.61 ± 0.07) × 10^3^ c
Δ*G*_1_ (cal·mol^−1^)	-	(−1.22 ± 0.04) × 10^4^ a	(−1.20 ± 0.03) × 10^4^ a	(−1.06 ± 0.29) × 10^4^ a
−T·Δ*S*_1_ (cal·mol^−1^)	-	(−2.16 ± 0.10) × 10^5^ c	(−1.49 ± 0.10) × 10^5^ b	(−4.01 ± 2.98) × 10^3^ a
**Set 2**	**ME**	**MMvE (E)**	**MMvE (Mv)**	**MMv_500_**
*K*_2_ (M^−1^)	-	(6.06 ± 0.14) × 10^8^ a	(3.16 ± 0.10) × 10^8^ b	(9.49 ± 0.76) × 10^4^ c
Δ*H*_2_ (cal·mol^−1^)	-	(−2.76 ± 0.03) × 10^5^ b	(−3.57 ± 0.18) × 10^5^ c	(−5.66 ± 0.12) × 10^3^ a
Δ*G*_2_ (cal·mol^−1^)	-	(−1.20 ± 0.03) × 10^4^ b	(−1.16 ± 0.04) × 10^4^ b	(−6.78 ± 0.54) × 10^3^ a
−T·Δ*S*_2_ (cal·mol^−1^)	-	(2.64 ± 0.03) × 10^5^ b	(3.46 ± 0.18) × 10^5^ a	(−1.12 ± 0.66) × 10^3^ c

MMvE (E): the interaction parameters of E in the ternary system; MMvE (Mv): the interaction parameters of Mv in the ternary system. Different letters in each row indicate statistical differences (*p* < 0.05) according to post hoc Tukey test.

## Data Availability

The data used to support the findings of this study can be made available by the corresponding author upon request.

## Abbreviations

Catechin (C), epicatechin (E), malvidin 3-*O*-glucoside (Mv), mucin (M), mucin:catechin binary interaction (MC), mucin:epicatechin binary interaction (ME), mucin:malvidin 3-*O*-glucoside binary interaction (MMv), mucin:malvidin 3-*O*-glucoside:catechin ternary interaction (MMvC), mucin:malvidin 3-*O*-glucoside:epicatechin ternary interaction (MMvE), phenolic compounds (PC), quercetin 3-*β*-glucopyranoside (QG).
